# CaMKII T286 phosphorylation has distinct essential functions in three forms of long-term plasticity

**DOI:** 10.1016/j.jbc.2022.102299

**Published:** 2022-07-21

**Authors:** Sarah G. Cook, Nicole L. Rumian, K. Ulrich Bayer

**Affiliations:** Department of Pharmacology, University of Colorado - Anschutz Medical Campus, Aurora, USA

**Keywords:** synaptic plasticity, long-term potentiation (LTP), long-term depression (LTD), Ca^2+^/calmodulin-dependent protein kinase II (CaMKII), N-methyl-D-aspartate receptor (NMDA receptor, NMDAR), metabotropic glutamate receptor (mGluR), hippocampus, neurons, ACSF, artificial cerebral spinal fluid, CaMKII, Ca^2+^/calmodulin-dependent protein kinase II, cLTD, chemical LTD, cLTP, chemical LTP, DHPG, (S)-3,5-dihydroxyphenylglycine, DIV, days *in vitro*, LTD, long-term depression, LTP, long-term potentiation, mGluR, metabotropic glutamate receptor, NMDAR, NMDA-type glutamate receptor

## Abstract

The Ca^2+^/calmodulin-dependent protein kinase II (CaMKII) mediates long-term potentiation or depression (LTP or LTD) after distinct stimuli of hippocampal NMDA-type glutamate receptors (NMDARs). NMDAR-dependent LTD prevails in juvenile mice, but a mechanistically different form of LTD can be readily induced in adults by instead stimulating metabotropic glutamate receptors (mGluRs). However, the role that CaMKII plays in the mGluR-dependent form of LTD is not clear. Here we show that mGluR-dependent LTD also requires CaMKII and its T286 autophosphorylation (pT286), which induces Ca^2+^-independent autonomous kinase activity. In addition, we compared the role of pT286 among three forms of long-term plasticity (NMDAR-dependent LTP and LTD, and mGluR-dependent LTD) using simultaneous live imaging of endogenous CaMKII together with synaptic marker proteins. We determined that after LTP stimuli, pT286 autophosphorylation accelerated CaMKII movement to excitatory synapses. After NMDAR-LTD stimuli, pT286 was strictly required for any movement to inhibitory synapses. Similar to NMDAR-LTD, we found the mGluR-LTD stimuli did not induce CaMKII movement to excitatory synapses. However, in contrast to NMDAR-LTD, we demonstrate that the mGluR-LTD did not involve CaMKII movement to inhibitory synapses and did not require additional T305/306 autophosphorylation. Thus, despite its prominent role in LTP, we conclude that CaMKII T286 autophosphorylation is also required for both major forms of hippocampal LTD, albeit with differential requirements for the heterosynaptic communication of excitatory signals to inhibitory synapses.

CaMKII is a central mediator of NMDAR-dependent long-term potentiation (LTP) and long-term depression (LTD) (1), two opposing forms of synaptic plasticity thought to mediate higher brain functions such as learning, memory, and cognition (2, 3, 4). Both LTP and LTD require the CaMKII autophosphorylation at T286 that generates Ca^2+^-independent autonomous CaMKII activity (5, 6). LTP additionally requires CaMKII binding to the NMDAR subunit GluN2B, which mediates the further accumulation of CaMKII at excitatory synapses (7, 8, 9, 10). By contrast, LTD instead requires additional inhibitory CaMKII autophosphorylation at T305/306 (11), which suppresses GluN2B binding and instead promotes CaMKII movement to inhibitory synapses, where it mediates inhibitory LTP (11, 12). NMDAR-dependent LTD is still detectable in adult hippocampus but is much more prevalent at juvenile stages (13, 14). However, robust LTD can still be induced in mature hippocampus by stimulation of group 1 mGluRs, *i.e.*, mGluR1 and 5 (4, 15, 16). A role of CaMKII also in this mGluR-dependent LTD has been suggested by at least three independent pharmacological studies (17, 18, 19). However, these pharmacological studies differed in the direction of the reported effect. Thus, it remained unclear if CaMKII promotes or inhibits mGluR-dependent LTD.

Here, we tested CaMKII functions in mGluR-LTD using genetic approaches. Our results show that, like NMDAR-dependent LTP and LTD, the mGluR-LTD requires the CaMKIIα isoform and its autophosphorylation at T286. Thus, we additionally compared the role of T286 phosphorylation in the CaMKII targeting to excitatory *versus* inhibitory synapses in response to these three plasticity stimuli. In NMDAR-LTD, the function of T286 phosphorylation is to enable induction of additional T305/306 phosphorylation, which is known to be required for the CaMKII movement to inhibitory synapses and for normal NMDAR-LTD (11). By contrast, mGluR-LTD did not require this additional inhibitory autophosphorylation. Thus, even though CaMKII T286 autophosphorylation has a longstanding prominent role in LTP, it is also required for both major forms of LTD in hippocampal neurons, albeit with differential requirements for the heterosynaptic communication of excitatory signals to inhibitory synapses.

## Results

### mGluR-LTD requires the CaMKIIα isoform

**Figure 1:**
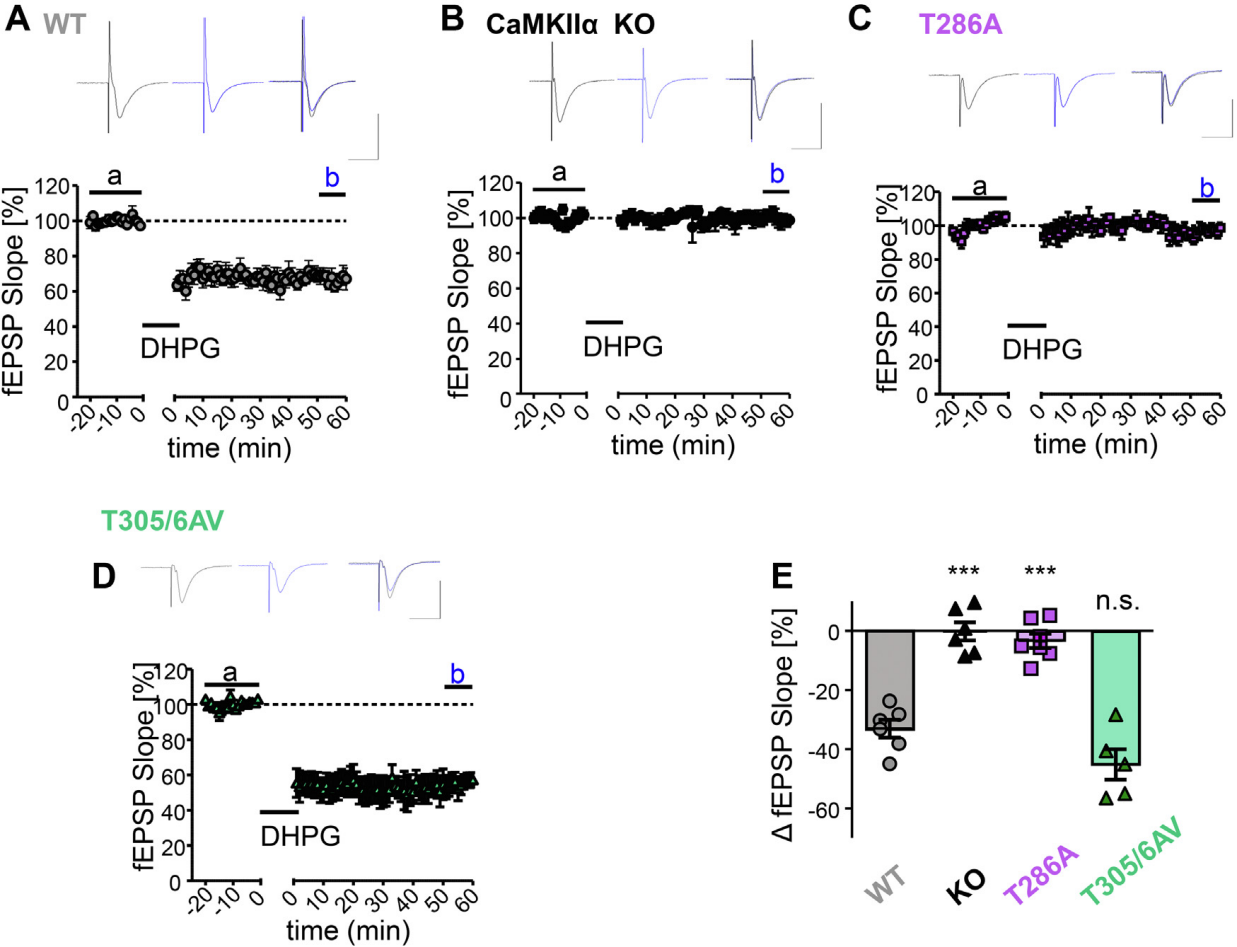
**mGluR-LTD requires the CaMKIIα isoform and its autophosphorylation at T286 but not T305/306.***A*, example traces and time course of synaptic response, measured by excitatory postsynaptic potential (EPSP) slope of the CA3-CA1 Schaffer collateral pathway, in wildtype hippocampal slices before and after chemical mGluR stimulation with DHPG (10 μM for 10 min). *B*, example traces and time course of synaptic response in CaMKIIα KO slices before and after the same DHPG treatment as in *A* show abolished mGluR-LTD. *C*, example traces and time course of synaptic response in T286A slices before and after DHPG treatment. *D*, example traces and time course of synaptic response in T305/306AV slices before and after stimulation with DHPG (10 μM for 10 min). *E*, quantification of the change in synaptic response (measured by EPSP slope) after DHPG stimulation in wildtype, CaMKIIα KO, T286A, and T305/306AV slices. Both CaMKII KO and T286A animals demonstrated severe impairments in DHPG-induced mGluR LTD when compared with wildtype animals while T305/306AV slices did not show any LTD deficit, indicating CaMKII and its T286 autophosphorylation are required for mGluR LTD (one-way ANOVA, Tukey’s post hoc test *versus* WT, ∗∗∗*p* < 0.001, n = 6, 6, 7, 5 slices).

As pharmacological studies yielded conflicting results about the role of CaMKII in mGluR-LTD, we decided to test CaMKII functions in this form of plasticity by genetic means. Here, group I mGluRs (*i.e.*, mGluR1 and mGluR5) were directly stimulated with (S)-3,5-dihydroxyphenylglycine (DHPG) (100 μM for 10 min). This treatment resulted in significant LTD at the CA3 to CA1 synapse in hippocampal slices from wildtype mice (Fig. 1*A*). Genetic knockout of the CaMKIIα isoform completely abolished this mGluR-LTD (Fig. 1*B*). Thus, like LTP and NMDAR-LTD (6, 20), normal mGluR-LTD specifically required the CaMKIIα isoform. Therefore, all additional CaMKII point mutations tested here were specifically in the α-isoform. Notably, the CaMKIIα isoform is exclusively expressed in neurons and is the major CaMKII isoform in mammalian brain, with ∼4-fold higher hippocampal expression than the next most prevalent isoform, CaMKIIβ (21).

### mGluR-LTD requires CaMKII autophosphorylation at T286 but not T305/306

In order to determine the specific mechanisms of CaMKII regulation required for mGluR-LTD, we next tested the effect of several point mutations of the endogenous CaMKIIα gene. The T286A mutation prevents the T286 autophosphorylation that generates Ca^2+^-independent autonomous kinase activity (22, 23, 24, 25). Like complete CaMKIIα knockout, this mutation completely abolished mGluR-LTD (Fig. 1*C*). Thus, like LTP and NMDAR-LTD (5, 6), mGluR-LTD specifically requires T286 autophosphorylation.

An additional mutant tested was T305/306AV, which prevents an inhibitory autophosphorylation at these residues that blocks Ca^2+^/CaM-binding and also curbs autonomous kinase activity (11, 24, 26, 27). In contrast to the T286A mutation, the T305/306AV mutation did not reduce mGluR-LTD at all (Fig. 1*D*). If any, the mGluR-LTD in the T305/306AV mice appeared to be slightly enhanced compared with the wildtype; however, this apparent enhancement was not statistically significant (Fig. 1*E*). Thus, in contrast to NMDAR-LTD (11) but like LTP (28), mGluR-LTD does not require the inhibitory autophosphorylation at T305/306.

The effects on mGluR-LTD of all CaMKII mutant mice tested here are summarized in Figure 1*E*. Overall, CaMKIIα and its T286 autophosphorylation is required for normal LTP, for NMDAR-LTD, and for mGluR-LTD. By contrast, additional T305/306 autophosphorylation is required only for normal NMDAR-LTD but not for LTP or mGluR-LTD.

### T286 autophosphorylation accelerates movement of endogenous CaMKII to excitatory synapses after LTP stimuli

**Figure 2:**
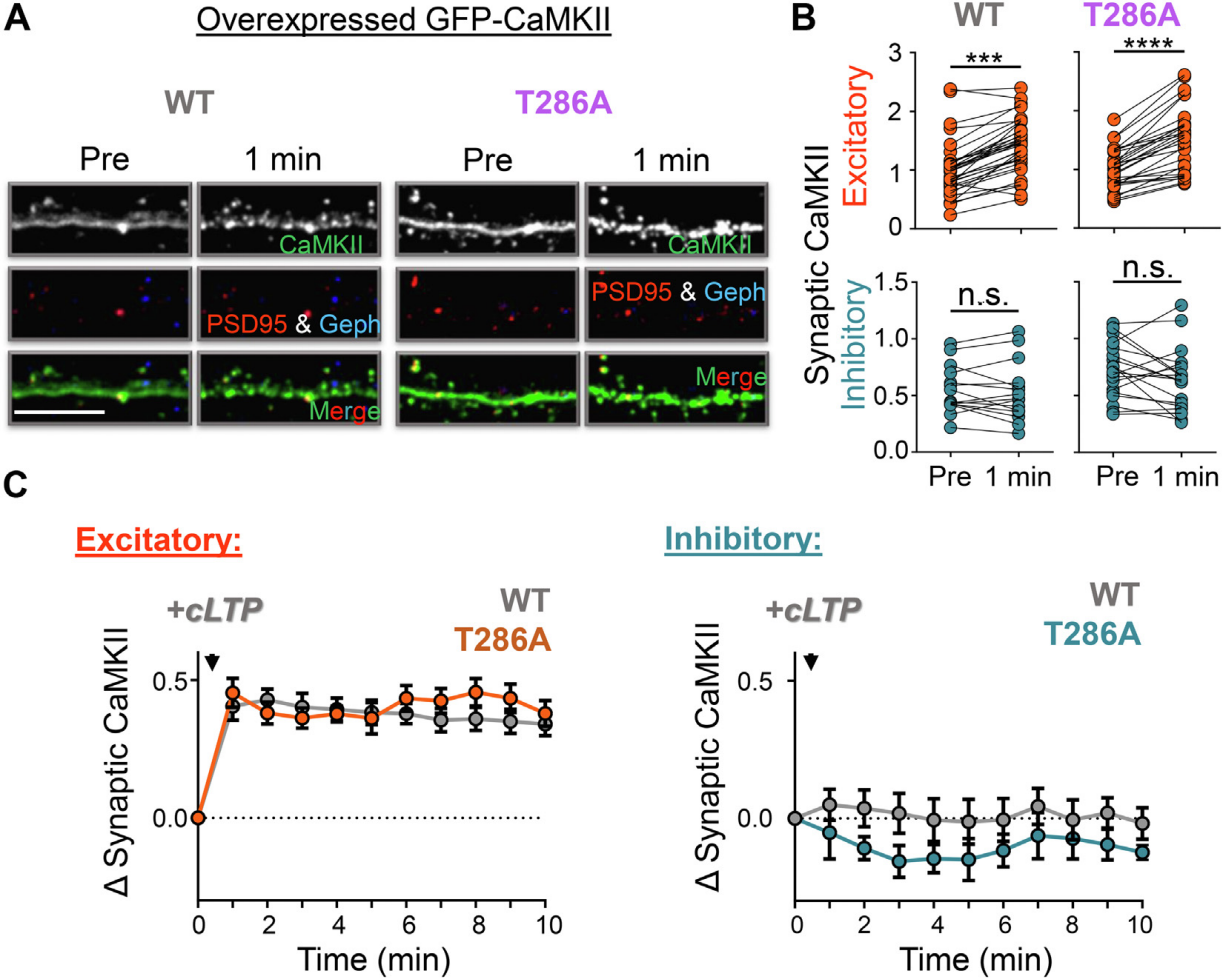
**T286A mutation does not affect the LTP-induced movement of overexpressed GFP-CaMKII.***A*, representative images of rat hippocampal neurons (DIV 14–17) expressing intrabodies for detection of PSD-95 (*red*) and gephyrin (*blue*) to label excitatory and inhibitory synapses, respectively, and overexpressing either CaMKII wildtype or T286A (*green*) before and 1 min following chemical NMDAR-LTP stimulation (cLTP; 100 μM glutamate/10 μM glycine, 1 min). The scale bar represents 10 μm. *B*, quantification of wildtype or T286A CaMKII at excitatory (*red*) and inhibitory (*blue*) synapses before and 1 min post cLTP stimulation. Both overexpressed wildtype and T286A CaMKII translocated to excitatory, but not inhibitory, synapses following cLTP treatment (paired *t* test, wildtype: ∗∗∗∗*p* < 0.0001, T286A: ∗∗∗*p* = 0.0003, n = 21, 12 neurons). *C*, full time course of wildtype (*gray*) and T286A CaMKII (*orange*) movement to excitatory and inhibitory synapses following cLTP stimulation indicating similar translocation dynamics for both constructs.

**Figure 3:**
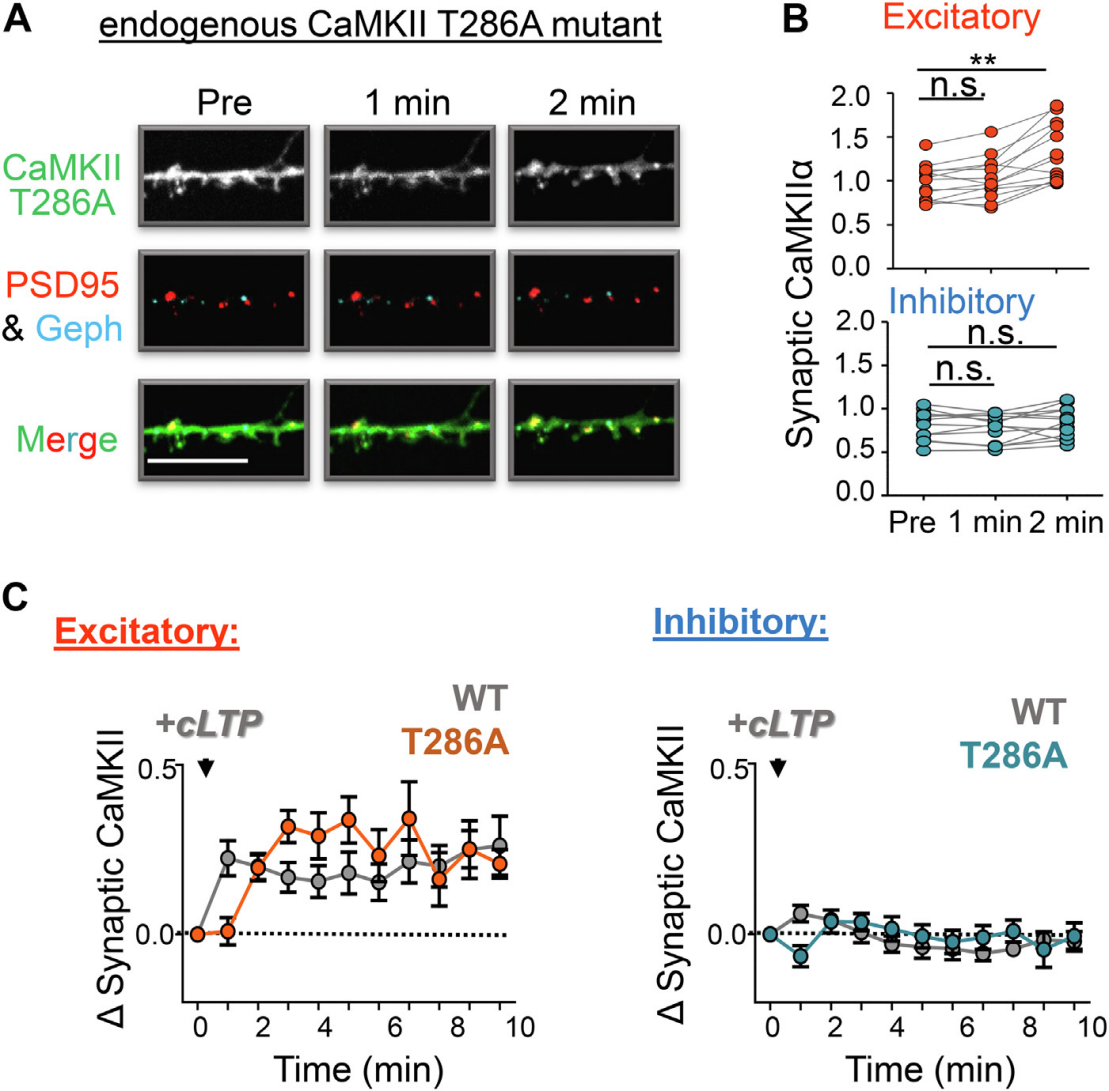
**LTP-induced movement of endogenous CaMKII to excitatory synapses is accelerated by T286 autophosphorylation.***A*, representative images of hippocampal neurons from CaMKII T286A mutant mice (DIV 14–17) expressing intrabodies against endogenous PSD-95 (*red*) and gephyrin (*blue*), to label excitatory and inhibitory synapses, respectively, and CaMKII (*green*) before, 1 min, and 2 min after cLTP treatment. The scale bar represents 10 μm. *B*, quantification of CaMKII T286A at excitatory (*red*) and inhibitory (*blue*) synapses before, 1 min, and 2 min post cLTP stimulation. Similar as described in wildtype neurons, endogenous CaMKII in hippocampal neurons from T286A mutant mice moved to excitatory but not inhibitory synapses in response to cLTP stimulation. However, the endogenous T286A mutant moved more slowly to excitatory synapses, as significant synaptic enrichment was seen only at 2 min but not at 1 min after cLTP stimuli. These results indicate T286 autophosphorylation accelerates LTP-induced CaMKII synaptic targeting (one-way ANOVA, Tukey’s post hoc test *versus* pre, ∗∗*p* = 0.0054, n = 13 neurons). *C*, full time course of T286A CaMKII movement to excitatory and inhibitory synapses following cLTP stimulation. For comparison, the previously described movement of wildtype CaMKII is illustrated in gray (11).

As T286 autophosphorylation is required for three distinct forms of long-term synaptic plasticity, we decided to determine how it affects CaMKII movement in hippocampal neurons in response to the different plasticity stimuli. It is known that T286 autophosphorylation is not strictly required for CaMKII movement to excitatory synapses in response to chemical LTP (cLTP) stimuli (29) or for Ca^2+^/CaM-induced binding to GluN2B *in vitro* (7). Indeed, the CaMKII movement induced by cLTP stimuli (1 min 100 μM glutamate in the presence of 10 μM glycine) was indistinguishable between overexpressed GFP-CaMKII wildtype and its T286A mutant (Fig. 2). However, when we instead monitored the movement of endogenous CaMKII with our intrabody method (11, 30), CaMKII movement was significantly faster in wildtype neurons compared with neurons from the mice with the T286A mutation in their endogenous CaMKIIα gene (Fig. 3). Thus, even though T286 autophosphorylation is not essential for the CaMKII movement to excitatory synapses that is required for normal LTP (8, 9), it significantly accelerates the process.

CaMKII movement to inhibitory synapses was not detected after cLTP stimuli, neither for overexpressed nor for endogenous CaMKII (Figs. 2 and 3), as expected based on our previous results (11, 30).

### T286 autophosphorylation is required for CaMKII movement to inhibitory synapses after NMDAR-LTD stimuli

**Figure 4:**
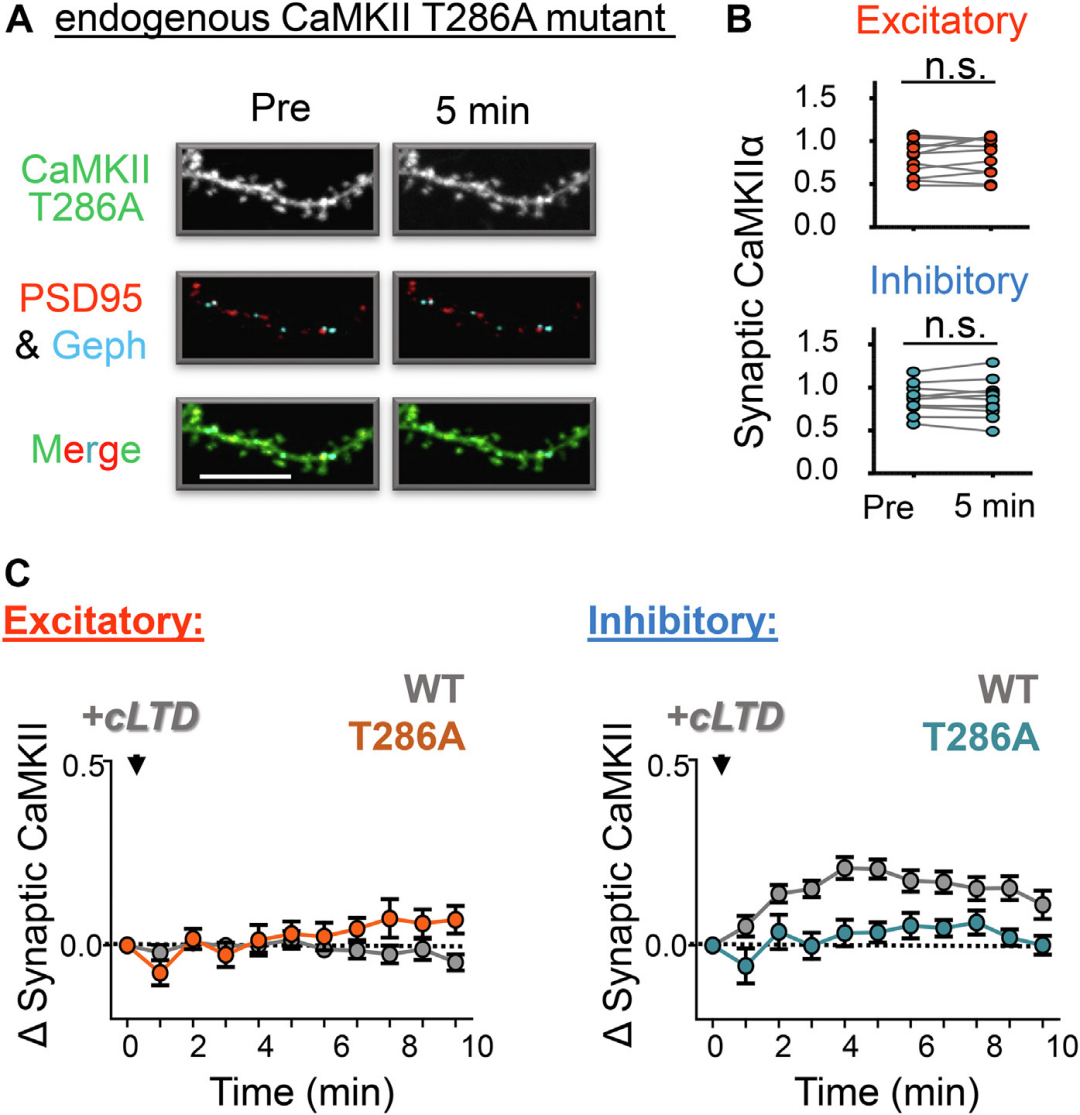
**CaMKII movement to inhibitory synapses in response to NMDAR-LTD stimuli requires T286 autophosphorylation.***A*, representative images of hippocampal neurons from CaMKII T286A mutant mice (DIV 14–17) expressing intrabodies against endogenous PSD-95 (*red*) and gephyrin (*blue*), to label excitatory and inhibitory synapses, respectively, and CaMKII (*green*) before and 5 min after chemical NMDAR-LTD stimuli (cLTD; 30 μM NMDA/10 μM CNQX/10 μM glycine, 1 min). The scale bar represents 10 μm. *B*, quantification of T286A CaMKII at excitatory (*red*) and inhibitory (*blue*) synapses prior to and 5 min post cLTD stimulation. In response to cLTD, endogenous T286A mutant CaMKII did not move to either excitatory or inhibitory synapses (in contrast to CaMKII wildtype, which has been described to move to inhibitory but not excitatory synapses in response to LTD). *C*, minimal movement of the T286A mutant to excitatory synapses (*red*) and lack of movement to inhibitory synapses (*blue*) following cLTD is further illustrated in a full time course up to 10 min after stimulation. For comparison, the previously described movement of wildtype CaMKII is illustrated in *gray* (11).

In contrast to cLTP stimuli, NMDAR-dependent chemical LTD (cLTD) stimuli (30 μM NMDA, 10 μM glycine, and 10 μM CNQX for 1 min) cause CaMKII movement to inhibitory synapses, and this movement strictly requires T305/306 autophosphorylation (11). An additional corequirement of T286 phosphorylation should be expected, because it is required for efficient T305/306 phosphorylation (11). Indeed, such requirement for T286 phosphorylation has been suggested by experiments with overexpressed GFP-CaMKII (31). Nonetheless, due to the discrepancies between movement of overexpressed and endogenous CaMKII in response to cLTP stimuli (see Figs. 2 and 3), we decided to examine the effect of T286A mutation also on endogenous CaMKII. As predicted, the T286A mutation completely abolished CaMKII movement to inhibitory synapses in response to NMDAR-dependent cLTD stimuli (Fig. 4). Thus, T286 autophosphorylation affects CaMKII movement to excitatory synapses in its temporal aspects, whereas it is absolutely required for any CaMKII movement to inhibitory synapses.

For excitatory synapses, no significant movement of the T286A mutant CaMKII was detected within 5 min after the cLTD stimulus (Fig. 4*A*, *B*), similar as described for the CaMKII wildtype. However, and in contrast to the CaMKII wildtype, a mild but significant movement was observed at 10 min after the cLTD stimulus (Fig. 4*C*; *p* < 0.01 in *t* test), consistent with T286A alleviating not only the subsequent T305/306 phosphorylation step that suppressed the movement but also the directly accelerating effect of the T286 phosphorylation (see Fig. 3).

### mGluR-LTD stimuli do not induce synaptic CaMKII movement

**Figure 5:**
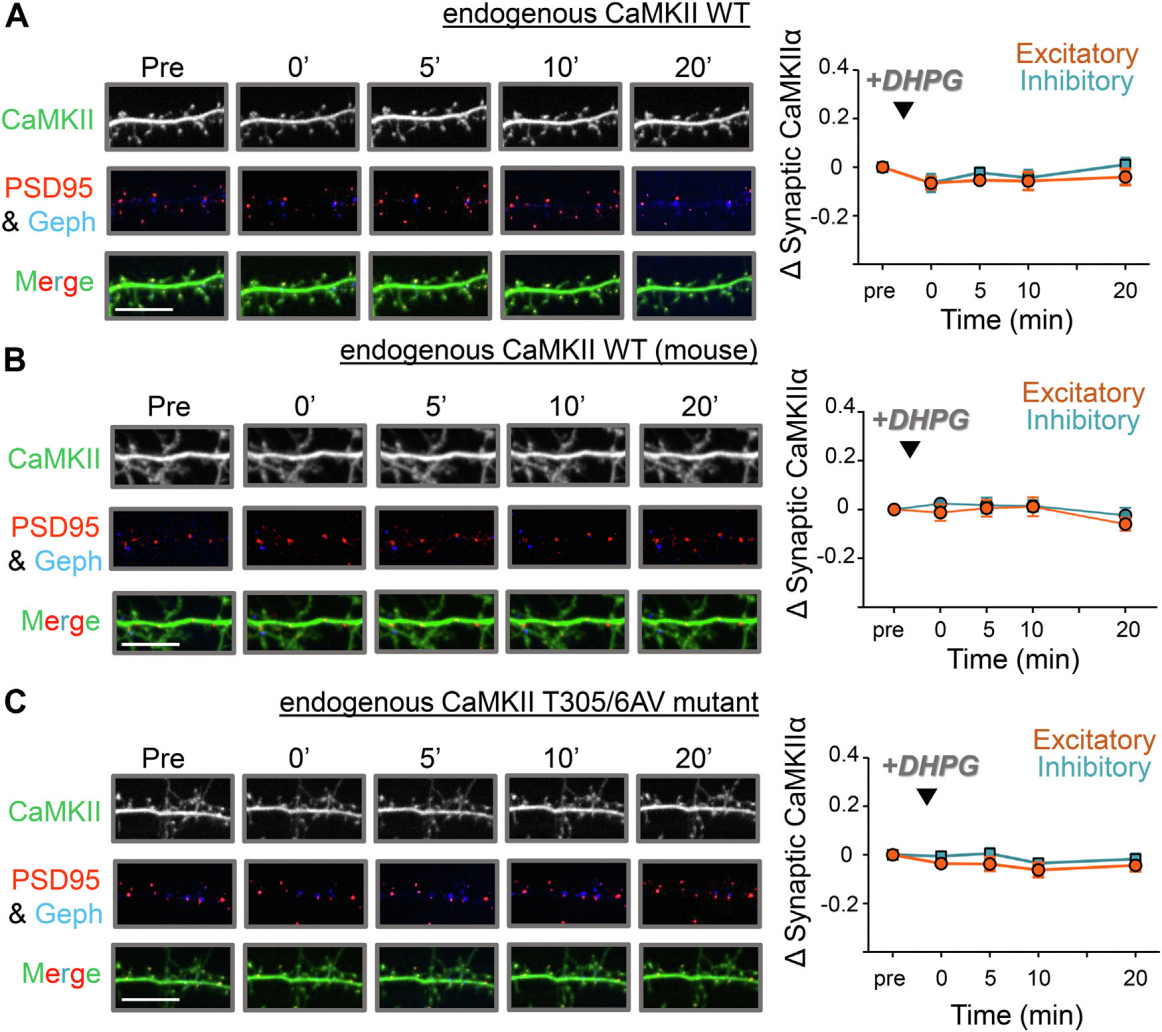
**mGluR stimulation does not promote CaMKII movement to synapses.***A*, representative images of rat hippocampal neurons (DIV 14–17) expressing intrabodies targeting endogenous CaMKII (*green*), as well as PSD-95 (*red*) and gephyrin (*blue*) to label excitatory and inhibitory synapses, respectively, before and 0, 5, 10, and 20 min after stimulation with DHPG (100 μM for 10 min). The scale bar represents 10 μM. The *left panel* shows a full time course quantification of endogenous wildtype CaMKII at excitatory (*red*) and inhibitory (*blue*) synapses prior to and 0, 5, 10, and 20 min post DHPG stimulation. No CaMKII movement to either excitatory or inhibitory synapses was found in response to DHPG stimulation (in contrast to NMDAR-LTD stimuli, where wildtype CaMKII has been described to move to inhibitory but not excitatory synapses). *B*, for comparison with mutant mice, hippocampal neurons of wildtype mice were subjected to the same experiments as the rat neurons shown in *A*, with essentially the same results. *C*, representative images of hippocampal neurons from CaMKII T305/306AV mutant mice (DIV 14–17) expressing intrabodies targeting endogenous CaMKII (*green*), as well as PSD-95 (*red*) and gephyrin (*blue*) to label excitatory and inhibitory synapses, respectively, before and 0, 5, 10, and 20 min after stimulation with DHPG (100 μM for 10 min). The scale bar represents 10 μM. The *left panel* shows a full time course quantification of endogenous T305/6AV CaMKII at excitatory (*red*) and inhibitory (*blue*) synapses prior to and 0, 5, 10, and 20 min post DHPG stimulation. Again, no movement of CaMKII to either excitatory or inhibitory synapses was found in response to DHPG stimulation.

As NMDAR-dependent cLTP or cLTD stimuli induce CaMKII movement to either excitatory or inhibitory synapses, respectively, we decided to examine if mGluR-LTD stimuli with 100 μM DHPG for 10 min also cause CaMKII movement. Endogenous CaMKII was monitored for 20 min after the DHPG stimulus in cultured hippocampal neurons, but no movement to either excitatory or inhibitory synapses was detected (Fig. 5*A,* *B*). After NMDAR-dependent cLTP stimuli, lack of CaMKII movement to excitatory synapses is mediated by active suppression mechanisms (11, 32), and the CaMKII T305/306AV mutation is sufficient to restore movement to excitatory synapses even after such cLTD stimuli (11). Thus, we also tested neurons from T305/306AV mutant mice for DHPG-induced CaMKII movement, but again, no movement was detected (Fig. 5*C*). This indicates either that the mechanisms for suppression of CaMKII movement differ in NMDAR- *versus* mGluR-LTD or that mGluR-LTD may not require such a suppression mechanism at all. Either scenario is consistent with the normal mGluR-LTD observed in hippocampal slices from the CaMKII T305/T306AV mutant mice (see Fig. 1, *D* and *E*).

Thus, overall, even though DHPG-induced mGluR-LTD does not induce any synaptic CaMKII movement, it does require the CaMKIIα isoform and its T286 phosphorylation (which accelerates the CaMKII movement to excitatory synapses after LTP and is absolutely required for the CaMKII movement to inhibitory synapses after NMDAR-LTD) but not its T305/306 phosphorylation (which is required for the specific suppression of CaMKII movement to excitatory synapses during NMDAR-LTD).

## Discussion

CaMKII is a central regulator of synaptic plasticity and well established to mediate both NMDAR-dependent LTP and LTD (for review see (1). Here, we demonstrate an additional requirement for CaMKII also in mGluR-LTD. Like NMDAR-LTP, but in contrast to NMDAR-LTD, this mGluR-LTD is robustly induced not only in young but also in mature animals. Like both forms of NMDAR-dependent plasticity (5, 6, 20), the mGluR-LTD required the CaMKIIα isoform and its autophosphorylation at T286. However, in contrast to NMDAR-LTD (11), the mGluR-LTD did not require additional autophosphorylation at T305/306. Consistent with this electrophysiological observation in hippocampal slices, imaging in hippocampal neurons showed that mGluR-LTD did not require T305/306 phosphorylation for suppression of CaMKII movement to excitatory synapses. This CaMKII movement is required for normal NMDAR-LTP (8, 9) and has to be actively suppressed during NMDAR-LTD (11, 32). If any active suppression of CaMKII movement is also required for mGluR-LTD, the mechanism must differ from NMDAR-LTD, as the latter requires T305/306 phosphorylation (11) whereas our results show that the former does not.

T286 phosphorylation is required for all three forms of long-term synaptic plasticity, but the time course of phosphorylation appears to differ. Phosphorylation at T286 generates Ca^2+^-independent “autonomous” activity and LTP stimuli had been proposed to cause a self-perpetuated increase in T286 phosphorylation. However, T286 autophosphorylation occurs between two different subunits of the 12meric CaMKII holoenzyme and requires Ca^2+^/CaM binding to both subunits (33, 34), and this mechanism does not support the originally proposed perpetuation mechanism. In addition, imaging experiments indicated that, while T286 autophosphorylation indeed extends the CaMKII activation state after LTP-stimuli, this was only on a time scale of less than 2 min (35). Nonetheless, the fast reversal of T286 phosphorylation after LTP remained somewhat controversial, in part because the imaging experiments did not directly assess T286 phosphorylation (for review see (36)). However, one of our recent studies directly compared T286 phosphorylation after NMDAR-dependent LTP *versus* LTD and indicated that LTP stimuli caused a larger increase in T286 phosphorylation but that this increase was fully reversed within 5 min (11). After NMDAR-LTD, the increase in T286 phosphorylation also appeared to be immediate but was then maintained longer (11). By contrast, the mGluR-LTD stimulus caused a delayed increase in T286 phosphorylation that was not apparent immediately but was significant after 5 min (18).

The different temporal pattern of T286 phosphorylation may help to enable the opposing downstream effects of CaMKII in the different forms of long-term plasticity. LTP additionally requires CaMKII binding to GluN2B (8, 9), whereas NMDAR-LTD instead additionally requires T305/306 autophosphorylation (11). Notably, GluN2B binding and T305/306 phosphorylation are both promoted by T286 phosphorylation (7, 37) but then mutually inhibit each other (7, 12). Thus, the different extent and time course of T286 phosphorylation may decide which one of the two downstream events after T286 phosphorylation prevails, *i.e.*, the GluN2B binding that is required for normal LTP or the T305/306 phosphorylation that is required for normal NMDAR-LTD. The mGluR-LTD appears to require neither of these two mechanisms: mGluR-LTD did not cause a GluN2B-mediated CaMKII accumulation in hippocampal neuron, and CaMKII phosphorylation at T305/306 was also not required, neither for the suppression of movement nor for the mGluR-LTD detected in hippocampal slices. While mGluR-LTD was expected to not require the GluN2B binding, lack of requirement of pT305/306 was somewhat more surprising. In NMDAR-LTD, such additional T305/306 phosphorylation is required to suppress CaMKII movement to excitatory synapses and instead direct CaMKII movement to inhibitory synapses, where it then induces inhibitory LTP (11). Consistent with a lack of T305/306 phosphorylation after mGluR-LTD, no CaMKII movement to inhibitory synapses was observed during this form of plasticity. Thus, whereas mGluR- and NMDAR-LTD both decrease the strength of excitatory synapses, only NMDAR-LTD stimuli appear to elicit additional heterosynaptic communication to inhibitory synapses.

The Ca^2+^/CaM-induced CaMKII binding to GluN2B appears to be both necessary and sufficient for the further accumulation of CaMKII at excitatory synapses during LTP (7, 9). However, CaMKII can bind to various other synaptic proteins (38, 39, 40, 41), including mGluR1 and mGluR5. Interestingly, Ca^2+^/CaM stimulates CaMKII binding to mGluR1 but disrupts binding to mGluR5, whereas CaMKII T286 autophosphorylation may increase binding to both (42, 43, 44). CaMKII binding and phosphorylation can regulate these mGluRs, but any potential contribution to the mGluR-LTD studied here is currently unknown. Notably, even though normal NMDAR-LTP requires CaMKII binding to GluN2B, the opposing NMDAR-LTD does not (9, 32). Furthermore, mGluR-LTD stimuli did not induce any detectable synaptic CaMKII movement. These parallels and observation do not rule out a function of CaMKII binding to mGluRs in mGluR-LTD. However, a direct function of CaMKII/mGluR binding in mGluR-LTD seems less conceivable than a more indirect function in mGluR metaplasticity, *i.e.*, in mediating signaling that modulates induction of subsequent mGluR-LTD. It will be interesting to elucidate the possible involvement of CaMKII protein–protein interactions in regulating different functions of mGluR-mediated plasticity, based on the requirement of CaMKII and its autophosphorylation at T286 but not T305/306 that was demonstrated here.

## Experimental procedures

### Material availability

Requests for resources, reagents, or questions about methods should be directed to K. Ulrich Bayer (ulli.bayer@cuanschutz.edu). This study did not generate new unique reagents.

### Experimental animals

All animal procedures were approved by the University of Colorado Institutional Animal Care and Use Committee (IACUC) and carried out in accordance with National Institutes of Health best practices for animal use. The University of Colorado Anschutz Medical Campus is accredited by the Association for Assessment and Accreditation of Laboratory Animal Care, International (AAALAC). All animals were housed in ventilated cages on a 12 h light/12 h dark cycle and were provided *ad libitum* access to food and water. Mixed sex wildtype or mutant mouse littermates (on a C57BL/6 background) from heterozygous breeder pairs (8–12 weeks old) were used for slice electrophysiology and biochemistry. Mixed sex pups from homozygous mice (P1-2) or Sprague-Dawley rats (P0, Charles River) were used to prepare dissociated hippocampal cultures for imaging. The mutant mice used here were described previously: the CaMKIIα knockout line used here was made in house (6); theT286A line was kindly provided by Ryohei Yasuda with kind permission from Karl Peter Giese (5); the T305/306AV mice were kindly provided by Ype Elgersma (28). In the T286A and in the T305/306AV mice, the mutations were introduced into the endogenous CaMKIIα gene (5, 28) and both lines have been used in the laboratory before (6, 11).

### Mouse hippocampal slice preparation

Isoflurane anesthetized mice were rapidly decapitated, and the brain was dissected in ice-cold high-sucrose solution containing (in mM): 220 sucrose, 12 MgSO4, 10 glucose, 0.2 CaCl2, 0.5 KCl, 0.65 NaH2PO4, 13 NaHCO3, and 1.8 ascorbate. Whole and CA1 mini hippocampal slices (400 μm) were made using a tissue chopper (McIlwain) with CA1 mini-slice preparation requiring additional cuts as described (11). Slices were transferred into 32 °C artificial cerebral spinal fluid (ACSF) containing (in mM): 124 NaCl, 2 KCl, 1.3 NaH2 PO4, 26 NaHCO3, 10 glucose, 2 CaCl2, 1 MgSO4, and 1.8 ascorbate and recovered in 95% O2/5% CO2 for at least 1.5 h before experimentation.

### Primary rat and mouse hippocampal culture

Primary hippocampal neurons were cultured as described (45) and imaged after 14 to 17 days *in vitro* (DIV14–17). Pups were decapitated and hippocampi were dissected and incubated in dissociation solution (7 ml HBSS buffered saline, 150 μl 100 mM CaCl2, 10 μl 1M NaOH, 10 μl 500 mM EDTA, 200 units Papain [Worthington]) at 25 °C for 1 h (rat) or 30 min (mice). Hippocampi were then washed 5× with plating medium (Dulbecco's modified Eagle's medium, fetal bovine serum, 50 units/ml Penn/strep, 2 mM L-glutamine, filter sterilized) and manually dissociated and counted using a hemocytometer. Dissociated neurons were plated on poly-D-lysine (0.1 mg/ml in 1 M borate buffer: 3.1 g boric acid, 4.75 g borax, in 1 L deionized H20, filter sterilized) and laminin (0.01 mg/ml in PBS)-coated 18-mm glass coverslips in 12-well plates at a density of 75,000 to 100,000 (rat) or 150,000 to 200,000 (mice) neurons per well in plating medium and maintained at 37 °C with 5% CO2. After 1 day *in vitro* (DIV 1), the medium was switched to 100% feeding medium (Neurobasal-A, B27 supplements, and 2 mM L-glutamine, filter sterilized). At DIV 5, 50% of medium was replaced with fresh neuron feeding medium and treated with FDU (70 μM 5-fluoro-2′-deoxyuridine/140 μM uridine) to suppress glial growth by halting mitosis. At 12 to 14 DIV, neurons were transfected with the intrabodies using a 1:1:1 ratio, at a concentration of 1 μg total DNA/well, using Lipofectamine 2000 (2.5 μl/well, Invitrogen) according to the manufacturer’s recommendations.

### Extracellular field recordings

All recordings and analysis were performed blind to genotype. For electrical slice recording experiments, a glass micropipette (typical resistance 0.4–0.8 MΩ when filled with ACSF) was used to record field excitatory postsynaptic potentials from the CA1 dendritic layer in response to stimulation in the Schaffer collaterals at the CA2 to CA1 interface using a tungsten bipolar electrode. Slices were continually perfused with 30.5 ± 0.5 °C ACSF at a rate of 3.5 ± 0.5 ml/min during recordings. Stimuli were delivered every 20 s and three responses (1 min) were averaged for analysis. Data were analyzed using WIN LTP (46) with slope calculated as the initial rise from 10 to 60% of response peak. Input/output (I/O) curves were generated by increasing the stimulus intensity at a constant interval until a maximum response or population spike was noted to determine stimulation that elicits 40 to 70% of maximum slope. Slope of I/O curve was calculated by dividing the slope of response (mV/ms) by the fiber volley amplitude (mV) for the initial linear increase. Paired-pulse recordings (50 ms interpulse interval) were acquired from 40% max slope, and no differences in presynaptic facilitation were seen in mutant slices. A stable baseline was acquired for a minimum of 20 min at 70% maximum slope prior to mGluR-LTD induction using 100 μM DHPG for 10 min. Slices were perfused for the remainder of the recording with ACSF containing an NMDAR-antagonist (50 μM APV). Change in slope was calculated as a ratio of the average slope of the 20 min baseline (prior to stimulation).

### Chemical LTP and LTD stimulation

NMDAR-dependent LTP (NMDAR-LTP) was chemically induced using 100 μM glutamate and 10 μM glycine for 1 min. NMDAR-dependent LTD (NMDAR-LTD) was chemically induced with 30 μM NMDA, 10 μM glycine, and 10 μM CNQX for 1 min. mGluR-dependent LTD (mGluR-LTD) was induced with 100 μM DHPG for 10 min. All treatments were followed by 5× washout in fresh ACSF. For imaging and biochemical experiments, quantifications demonstrate the change from 1 min prestimulation and 1 min (cLTP) or 5 min (cLTD) post wash out unless otherwise specified.

### Imaging acquisition and analysis

All microscopic imaging was performed using a 100 × 1.4NA objective on a Zeiss Axiovert 200 M (Carl Zeiss) controlled by SlideBook software (Intelligent Imaging Innovations). All imaging analysis was completed using SlideBook software. All representative images were prepared using Fiji software (ImageJ, NIH). For all imaging experiments, focal plane z stacks (0.3-μm steps; over 1.8–2.4 μm) were acquired and deconvolved to reduce out-of-focus light. 2D maximum intensity projection images were then generated and analyzed by an experimenter blinded to experimental conditions. During image acquisition, neurons were maintained at 34 °C in ACSF solution containing (in mM): 130 NaCl, 5 KCl, 10 Hepes pH 7.4, 20 glucose, 2 CaCl2, and 1 MgCl2, adjusted to proper osmolarity with sucrose. After baseline imaging and cLTP or cLTD treatment, neurons were imaged once per minute for 10 min to limit the effects of photobleaching.

Hippocampal neurons were selected based on pyramidal shaped soma and presence of spiny apical dendrites, and tertiary dendritic branches were selected for analysis to maintain consistency. Images were analyzed at 1 min before stimulation and 1 min (after cLTP), 5 min (after cLTD), and 10 min (after DHPG) after wash out. 2D maximum intensity projection images were then generated and analyzed by an experimenter blinded to condition using Slidebook 6.0 software. To analyze endogenous YFP-intrabody-labeled CaMKII and overexpressed GFP-labeled CaMKII, the mean YFP or GFP intensity (CaMKII) at excitatory (PSD-95) and inhibitory (gephyrin) synapses was quantified. PSD-95 and gephyrin threshold masks were defined using the mean intensity of mCh or mTurquois plus two standard deviations. Synaptic CaMKII was then calculated using the mean YFP or GFP intensity at PSD-95 or gephyrin puncta masks divided by the mean intensity of a line drawn in the dendritic shaft. Changes in CaMKII synaptic accumulation were determined by dividing the net change in CaMKII at PSD-95 or gephyrin puncta-to-shaft ratio by the prestimulation puncta-to-shaft ratio.

### Quantification and statistical analysis

Data are shown as mean ± SEM, and all imaging and Western blot quantification is normalized to control average set to 1. Statistical significance and sample size (n) are indicated in the figure legends. Data obtained from imaging experiments were obtained using SlideBook 6.0 software (3i) and analyzed using Prism (GraphPad) software. All data met parametric conditions, as evaluated by a Shapiro–Wilk test for normal distribution and a Brown–Forsythe test (three or more groups) or an F-test (two groups) to determine equal variance. Comparisons between two groups were analyzed using unpaired, two-way Student’s *t* tests. Comparisons between pre- and posttreatment images from the same cells were analyzed using paired, two-way Student’s *t* tests. Comparisons between three or more groups were done by one-way ANOVA with Tukey’s post hoc analysis. Comparisons between three or more groups with two independent variables were accessed using a two-way ANOVA to determine whether there is an interaction and/or main effect between the variables. Statistical significance is indicated, including by ∗*p* < 0.05; ∗∗*p* < 0.01; ∗∗∗*p* < 0.001, ∗∗∗∗ *p* < 0.0001.

## Data and code availability

The datasets generated during this study are available through Mendeley. No original code was generated during this study.

## Conflict of interest

K. U. B. is cofounder and board member of Neurexis Therapeutics.

## References

[1] Bayer K.U., Schulman H. (2019). CaM kinase: still inspiring at 40. Neuron.

[2] Martin S.J., Grimwood P.D., Morris R.G. (2000). Synaptic plasticity and memory: an evaluation of the hypothesis. Annu. Rev. Neurosci..

[3] Malenka R.C., Bear M.F. (2004). LTP and LTD: an embarrassment of riches. Neuron.

[4] Collingridge G.L., Peineau S., Howland J.G., Wang Y.T. (2010). Long-term depression in the CNS. Nat. Rev. Neurosci..

[5] Giese K.P., Fedorov N.B., Filipkowski R.K., Silva A.J. (1998). Autophosphorylation at Thr286 of the alpha calcium-calmodulin kinase II in LTP and learning. Science.

[6] Coultrap S.J., Freund R.K., O'Leary H., Sanderson J.L., Roche K.W., Dell'Acqua M.L. (2014). Autonomous CaMKII mediates both LTP and LTD using a mechanism for differential substrate site selection. Cell Rep..

[7] Bayer K.U., De Koninck P., Leonard A.S., Hell J.W., Schulman H. (2001). Interaction with the NMDA receptor locks CaMKII in an active conformation. Nature.

[8] Barria A., Malinow R. (2005). NMDA receptor subunit composition controls synaptic plasticity by regulating binding to CaMKII. Neuron.

[9] Halt A.R., Dallpiazza R.F., Zhou Y., Stein I.S., Qian H., Juntti S. (2012). CaMKII binding to GluN2B is critical during memory consolidation. EMBO J..

[10] Incontro S., Diaz-Alonso J., Iafrati J., Vieira M., Asensio C.S., Sohal V.S. (2018). The CaMKII/NMDA receptor complex controls hippocampal synaptic transmission by kinase-dependent and independent mechanisms. Nat. Commun..

[11] Cook S.G., Buonarati O.R., Coultrap S.J., Bayer K.U. (2021). CaMKII holoenzyme mechanisms that govern the LTP *versus* LTD decision. Sci. Adv..

[12] Barcomb K., Buard I., Coultrap S.J., Kulbe J.R., O'Leary H., Benke T.A. (2014). Autonomous CaMKII requires further stimulation by Ca2+/calmodulin for enhancing synaptic strength. FASEB J..

[13] Dudek S.M., Bear M.F. (1992). Homosynaptic long-term depression in area CA1 of hippocampus and effects of N-methyl-D-aspartate receptor blockade. Proc. Natl. Acad. Sci. U. S. A..

[14] Dudek S.M., Bear M.F. (1993). Bidirectional long-term modification of synaptic effectiveness in the adult and immature hippocampus. J. Neurosci..

[15] Luscher C., Huber K.M. (2010). Group 1 mGluR-dependent synaptic long-term depression: Mechanisms and implications for circuitry and disease. Neuron.

[16] Reiner A., Levitz J. (2018). Glutamatergic signaling in the central nervous system: ionotropic and metabotropic receptors in concert. Neuron.

[17] Schnabel R., Palmer M.J., Kilpatrick I.C., Collingridge G.L. (1999). A CaMKII inhibitor, KN-62, facilitates DHPG-induced LTD in the CA1 region of the hippocampus. Neuropharmacology.

[18] Mockett B.G., Guevremont D., Wutte M., Hulme S.R., Williams J.M., Abraham W.C. (2011). Calcium/calmodulin-dependent protein kinase II mediates group I metabotropic glutamate receptor-dependent protein synthesis and long-term depression in rat hippocampus. J. Neurosci..

[19] Bernard P.B., Castano A.M., Bayer K.U., Benke T.A. (2014). Necessary, but not sufficient: Insights into the mechanisms of mGluR mediated long-term depression from a rat model of early life seizures. Neuropharmacology.

[20] Silva A.J., Stevens C.F., Tonegawa S., Wang Y. (1992). Deficient hippocampal long-term potentiation in a-calcium-calmodulin kinase II mutant mice. Science.

[21] Cook S.G., Bourke A.M., O'Leary H., Zaegel V., Lasda E., Mize-Berge J. (2018). Analysis of the CaMKIIalpha and beta splice-variant distribution among brain regions reveals isoform-specific differences in holoenzyme formation. Sci. Rep..

[22] Coultrap S.J., Buard I., Kulbe J.R., Dell'Acqua M.L., Bayer K.U. (2010). CaMKII autonomy is substrate-dependent and further stimulated by Ca2+/calmodulin. J. Biol. Chem..

[23] Miller S.G., Kennedy M.B. (1986). Regulation of brain type II Ca2+/calmodulin-dependent protein kinase by autophosphorylation: A Ca2+-triggered molecular switch. Cell.

[24] Lou L.L., Schulman H. (1989). Distinct autophosphorylation sites sequentially produce autonomy and inhibition of the multifunctional Ca2+/calmodulin-dependent protein kinase. J. Neurosci..

[25] Colbran R.J., Smith M.K., Schworer C.M., Fong Y.L., Soderling T.R. (1989). Regulatory domain of calcium/calmodulin-dependent protein kinase II. Mechanism of inhibition and regulation by phosphorylation. J. Biol. Chem..

[26] Colbran R.J., Soderling T.R. (1990). Calcium/calmodulin-independent autophosphorylation sites of calcium/calmodulin-dependent protein kinase II. Studies on the effect of phosphorylation of threonine 305/306 and serine 314 on calmodulin binding using synthetic peptides. J. Biol. Chem..

[27] Hanson P.I., Schulman H. (1992). Inhibitory autophosphorylation of multifunctional Ca2+/calmodulin-dependent protein kinase analyzed by site-directed mutagenesis. J. Biol. Chem..

[28] Elgersma Y., Fedorov N.B., Ikonen S., Choi E.S., Elgersma M., Carvalho O.M. (2002). Inhibitory autophosphorylation of CaMKII controls PSD association, plasticity, and learning. Neuron.

[29] Shen K., Meyer T. (1999). Dynamic control of CaMKII translocation and localization in hippocampal neurons by NMDA receptor stimulation. Science.

[30] Cook S.G., Goodell D.J., Restrepo S., Arnold D.B., Bayer K.U. (2019). Simultaneous live-imaging of multiple endogenous proteins reveals a mechanism for Alzheimer’s-related plasticity impairment. Cell Rep..

[31] Marsden K.C., Shemesh A., Bayer K.U., Carroll R.C. (2010). Selective translocation of Ca2+/calmodulin protein kinase IIalpha (CaMKIIalpha) to inhibitory synapses. Proc. Natl. Acad. Sci. U. S. A..

[32] Goodell D.J., Zaegel V., Coultrap S.J., Hell J.W., Bayer K.U. (2017). DAPK1 mediates LTD by making CaMKII/GluN2B binding LTP specific. Cell Rep..

[33] Hanson P.I., Meyer T., Stryer L., Schulman H. (1994). Dual role of calmodulin in autophosphorylation of multifunctional CaM kinase may underlie decoding of calcium signals. Neuron.

[34] Rich R.C., Schulman H. (1998). Substrate-directed function of calmodulin in autophosphorylation of Ca2+/calmodulin-dependent protein kinase II. J. Biol. Chem..

[35] Lee S.J., Escobedo-Lozoya Y., Szatmari E.M., Yasuda R. (2009). Activation of CaMKII in single dendritic spines during long-term potentiation. Nature.

[36] Lisman J., Yasuda R., Raghavachari S. (2012). Mechanisms of CaMKII action in long-term potentiation. Nat. Rev. Neurosci..

[37] Strack S., McNeill R.B., Colbran R.J. (2000). Mechanism and regulation of calcium/calmodulin-dependent protein kinase II targeting to the NR2B subunit of the N-methyl-D-aspartate receptor. J. Biol. Chem..

[38] Colbran R.J. (2004). Targeting of calcium/calmodulin-dependent protein kinase II. Biochem. J..

[39] Kim K., Saneyoshi T., Hosokawa T., Okamoto K., Hayashi Y. (2016). Interplay of enzymatic and structural functions of CaMKII in long-term potentiation. J. Neurochem..

[40] Hell J.W. (2014). CaMKII: claiming center stage in postsynaptic function and organization. Neuron.

[41] Bayer K.U., Schulman H. (2001). Regulation of signal transduction by protein targeting: the case for CaMKII. Biochem. Biophys. Res. Commun..

[42] Jin D.Z., Guo M.L., Xue B., Mao L.M., Wang J.Q. (2013). Differential regulation of CaMKIIalpha interactions with mGluR5 and NMDA receptors by Ca(2+) in neurons. J. Neurochem..

[43] Jin D.Z., Guo M.L., Xue B., Fibuch E.E., Choe E.S., Mao L.M. (2013). Phosphorylation and feedback regulation of metabotropic glutamate receptor 1 by calcium/calmodulin-dependent protein kinase II. J. Neurosci..

[44] Marks C.R., Shonesy B.C., Wang X., Stephenson J.R., Niswender C.M., Colbran R.J. (2018). Activated CaMKIIalpha binds to the mGlu5 metabotropic glutamate receptor and modulates calcium mobilization. Mol. Pharmacol..

[45] Vest R.S., O'Leary H., Coultrap S.J., Kindy M.S., Bayer K.U. (2010). Effective post-insult neuroprotection by a novel Ca(2+)/calmodulin-dependent protein kinase II (CaMKII) inhibitor. J. Biol. Chem..

[46] Anderson W.W., Collingridge G.L. (2001). The LTP program: a data acquisition program for on-line analysis of long-term potentiation and other synaptic events. J. Neurosci. Met..

